# Recruitment and retention of tutors in problem-based learning: why teachers in medical education tutor

**Published:** 2013-03-31

**Authors:** Teresa Paslawski, Ramona Kearney, Jonathan White

**Affiliations:** 1 Department of Speech Language Pathology and Audiology, University of Alberta, Canada; 2 Department of Anesthesiology and Pain Medicine, University of Alberta, Canada; 3 Department of Surgery, University of Alberta, Canada

## Abstract

**Introduction:**

Problem-based learning (PBL) is resource-intensive, particularly as it relates to tutors for small group learning. This study explores the factors that contributed to tutor participation in PBL in a medical training program, examining tutor recruitment and retention within the larger scope of teacher satisfaction and motivation in higher education.

**Method:**

From 2007 to 2010, following the introduction of new PBL-based curriculum in undergraduate medical education, all faculty members serving as tutors were invited to attend an interview as part of this study. Semi-structured interviews approximately one hour in length were conducted with 14 individuals- 11 who had tutored in PBL within the Faculty of Medicine and Dentistry and 3 faculty members who had chosen not to participate in PBL. Thematic analysis was employed as the framework for analysis of the data.

**Results:**

Seven factors were identified as affecting recruitment and retention of tutors in the undergraduate medical education program.

**Discussion:**

We suggest that identification and strengthening of the factors that promote tutor recruitment and retention may serve to strengthen PBL initiatives and, furthermore, may increase our understanding of motivation by academics in other aspects of medical education.

## Introduction

Many medical schools world-wide have adopted problem-based learning (PBL) as an approach to training.^1^ PBL generally involves small groups of students who meet with a tutor to guide the process of discovery with respect to the learning objectives of a particular case. In contrast to the traditional lecture format, this small group-tutor organization is resource-intensive with respect to personnel. The need to have a tutor for each small group necessitates effective recruitment and retention of trained facilitators to contribute to PBL.

Approaches to tutor recruitment have included requiring participation by faculty members, or training students to serve as PBL facilitators.^2^–^4^ Farmer^5^ acknowledges that to obtain adequate numbers of tutors for PBL, faculty likely need to be encouraged to participate in PBL and cautions that there may be differences in the effectiveness of tutors who volunteer compared to those who are required to participate. Certainly, our own examination of the attitudes and beliefs of conscripted and volunteer PBL tutors^6^ indicates that there are differences between these two groups. In that survey, which involved 110 tutors, respondents were asked to identify themselves as volunteer participants in PBL tutoring or conscripted, and then were asked to respond to a series of questions about PBL. Of particular relevance to the current study, more conscripted tutors reported that they believed PBL would increase the amount of time they spent on education and that it would negatively affect their careers. Attitudes such as these would be expected to have an adverse effect on recruitment and retention of PBL facilitators. Finucane and colleagues^7^ acknowledge the challenge and importance of ensuring adequate numbers of tutors but further suggest that content knowledge and competence in the PBL process are also important to effective PBL delivery, and that experience is a desirable quality in a tutor.

Understanding the motivation of tutors who participate in PBL and the factors that contribute to satisfaction and dissatisfaction with respect to tutoring is central to improving approaches to tutor recruitment and, following training in PBL, retention of experienced tutors. In addition to attracting willing and able tutors to PBL, the value of understanding motivation in this group of academics may also have a larger impact. Rowley^8^ suggests that motivation in academia is a critical component in developing quality higher education, and several studies have indicated that there is a relationship between motivation and job satisfaction.^9^–^12^ Others have also called for more work exploring teachers’ conceptions of their roles as tutors in PBL.^13^

The literature on why teachers teach is largely focused on primary and secondary settings. Less is known about factors that contribute to the decision to teach in post-secondary institutions, despite the fact that teaching is part of the job requirement of the majority of faculty at post-secondary institutions. Ironically, academics are an “understudied occupational group”.^14^ As Gmelch and colleagues indicated, “We, as academics and researchers, willingly study other groups yet we seldom take time to look at our own profession”.^15^

In the absence of a significant literature on why university teachers teach, factors that influence academics’ motivations to teach are found embedded in the larger examination of occupational stress in universities.^14^, ^16^–^18^ This paper explores the factors that contribute to faculty members’ decisions regarding participation in a PBL initiative. Factors were identified through interviews with faculty members who served as tutors in a PBL program. These factors will be discussed in the context of current understandings of job satisfaction in universities.

### Background

A new curriculum based on the principles of problem-based learning (named “Discovery Learning” or DL) was implemented in the pre-clinical years (years 1 and 2) of the Doctor of Medicine program at the university in the fall of 2007. Faculty members from each department within the Faculty of Medicine and Dentistry were expected to participate in PBL and were required to attend two training workshops prior to tutoring. The first workshop was intended to introduce PBL as a learner-centered curriculum, to identify the structure of PBL being adopted by the Faculty and to define the role of the tutor in PBL. The second workshop was designed to develop facilitation skills, offering a hands-on experience of facilitation in PBL including a debriefing session. This second workshop also served to reinforce the format of each session with the students and the process of PBL, to encourage consistency among tutors.

In keeping with one approach to PBL indicating that tutors need not be experts in the subject area in order to be expert tutors,^19^ tutors were intentionally assigned to PBL courses outside of their area of expertise. Tutors were offered weekly group debriefing sessions facilitated by a PBL trainer during which time they were invited to discuss questions and concerns as well as to share insights regarding their PBL sessions.

This study examines why tutors in problem-based learning in a medical school participated in PBL and what factors contributed to their decisions regarding continuation of participation. The factors identified will be discussed in the context of the literature regarding teacher motivation, occupational stress and job satisfaction.

## Method

For three years following the introduction of the new curriculum, data were collected on tutor recruitment and retention. All faculty members serving as tutors were invited to attend an interview as part of this study. Semi-structured interviews approximately one hour in length were conducted. Interviews were recorded and transcribed by a professional transcriptionist for analysis. Identifiers were removed from the transcripts by the interviewer to protect the confidentiality of all study participants. With the exception of the interviewer who was a member of the research team, the identities of the participants were not known to the researchers. Thematic analysis was employed as the framework for analysis of the data. Two reviewers independently developed coding schemes based on themes within the narratives, then compared notes and came to consensus regarding themes and related factors. Quotes that most accurately reflected these themes were also identified. These themes were then discussed alongside the raw data by the research team and modified until consensus was achieved. Further discussion by the entire team resulted in agreement regarding interpretation of the data and identification of the factors.^20^

### Sample

In the first year of introduction of the new curriculum, 331 faculty members had their names put forward by Department Chairs to become tutors. Of these, 163 completed both training workshops (49%). Fifty six (17%) participated only in the second workshop based on previous training and experience in PBL (approved by the Director of Faculty Development for PBL). A total of 198 tutors (60%) participated in DL sessions in the first year of operation. In the second year of operation (2008–9), 79% of tutors volunteered to return and serve as a tutor again. In the third year of operation (2009–10), the return rate was 93%.

Semi-structured interviews (Appendix 1) were conducted with 14 individuals- 11 who had tutored in PBL within the Faculty of Medicine and Dentistry and 3 faculty members who had chosen not to participate in PBL. Of the 11 tutors, 6 continued to participate in PBL while 5 did not tutor beyond their initial experience. Five of the 14 tutors interviewed noted that they had been required to participate by their Department Chair.

## Results

The following seven factors were identified as affecting recruitment and retention of tutors in the undergraduate medical education program.

### 1. “Volunteer or Conscript?”

Seven of the eleven participants who were PBL tutors indicated that they were interested in PBL as an approach to teaching, noting that it was a teaching technique adopted by other medical schools. In the majority of cases, participants indicated previous involvement in PBL, with two respondents acknowledging that they had been trained using PBL and they wanted to “*see it from the other side*”. Tutors reflected that PBL “*offered an opportunity to make a different impact*”. Of the recommendations regarding tutor recruitment and retention, one of the most passionately debated was that of requiring individuals to tutor in PBL as opposed to identifying faculty members who agreed with this approach to teaching and learning.

### 2. Tutor Training

Tutor training was also specifically cued during the interview, to determine if this was considered a form of support. The majority of those interviewed believed that the training workshops were valuable. “*I think I’ve been trained appropriately*.” The standardization of the PBL sessions was commented on specifically by one respondent as being an important component. An extension of the training workshops were debriefing sessions held on a regular basis in conjunction with the PBL blocks that tutors were invited to attend. These too were recognized as “*a great idea*” and appreciated by those who chose to continue tutoring in PBL, though it was also noted that this was an additional time commitment, which added to the resource-intensiveness of PBL.

The second PBL tutor training session included a simulation of a PBL session, involving actual medical students who were familiar with PBL. The tutors-in-training were required to spend a short period of time (< 15 minutes) facilitating part of a case, observed by a PBL trainer and a small group of other tutors-in-training. After the simulation the small groups engaged in a debrief regarding the PBL process. One tutor commented that “*the simulation training was a brilliant idea*”. Conversely, two of the tutors responded negatively to the simulation component, indicating that it was “*unnerving*”, “*too artificial” and “counter-productive, it may have put people off*”. One of the tutors suggested that they would have preferred to “*observe an actual [PBL] session or two*” rather than engage in the simulation exercise.

### 3. Tutor Support

Administrative support services, specifically cued during the interviews, overlaps with time commitment and scheduling, discussed under factor 7, Barriers to Commitment. Participants indicated strongly that this was an important and highly positive factor affecting their participation in PBL. Respondents commented that the level of administrative support was “*superb*”, “*excellent*”, “*outstanding*” and “*very much appreciated*”. More specifically, respondents noted the positive attitude on the part of the administrative support staff as reflected in the high degree of flexibility, a willingness to accommodate tutors’ needs, and the “*ability to problem-solve, for example, concerns with students and who the facilitator should be connected to in order to handle the problem*”. In addition, tutors appreciated the quality of communication and organization which they indicated contributed to their feeling of being supported. The importance of administrative support was underscored by tutors who indicated, “*You need the support in order to be creative.*” And “*the attitude is so positive and so supportive and so encouraging that you want to work with these people*”.

### 4. “Content Expert or Expert Facilitator”

When the new curriculum was introduced, tutors were discouraged from tutoring in courses where they would be considered content experts. Response to this practice was met with mixed reviews. One tutor commented that because they were not an expert in the area, they had “*the feeling that [they weren’t] very helpful to the students*”. Another was concerned that it was “*unfair to the students*” that a non-expert was facilitating the small group discussion.

Conversely, others described a process of becoming comfortable with the role of facilitator as opposed to being an expert, *“[I experienced] initial frustration about not being allowed to teach…. finding the balance of being a guide without lecturing was a challenge*”. One respondent indicated that PBL was enjoyable because it was consistent with their philosophy of education. A benefit of non-experts as tutors identified by a non-clinical tutor was that *“[it] helps students understand how to relate to non-clinicians*”.

A positive view of the expert facilitator is described in the following observation:

“….Initially I felt somewhat intimidated by being asked to facilitate in an area that was remote from my clinical interests and from my recent clinical experiences…. But by virtue of diving in in a non-expert area, I got comfortable with that. The fact that I could be an effective tutor in those areas was helpful to me and, unexpectedly, it was actually quite rewarding and stimulating because it pushed me to think around other problems, other types of medical problems, and to re-invest in some of what I have already been involved in doing previously in terms of knowledge base. But also, it reinforced the importance of the common principles between common basic science principles between the different clinical blocks.”

Conversely, the tutorial experience was perceived so negatively by one respondent that they decided not to return to facilitate a second time. “*[It was] terrible. I didn’t enjoy it at all.*”

### 5. Feedback and Relationship Building

Built into the PBL sessions were opportunities to give and receive feedback, on the part of the tutor (to the students) and of the students (to the tutor). This was identified as being extremely important by the tutors.

“The other thing that assisted me in going forward was putting some emphasis really on getting to know the students. And getting the students to sort of loosen up and feel comfortable in the environment was very important. Giving them feedback and getting feedback from them was a very important part of the process.”

Giving and receiving feedback on a regular basis was seen as part of relationship building as well as a means of improving skills, the tutors felt they were “*learning with the students*”. “*Getting student feedback … more frequently was helpful so you could alter your approach to facilitation*”. “*Both faculty and students learn a lot from the process of getting/giving feedback*”. Two tutors remarked that because they developed relationships with the students in the PBL group, they recruited students into their labs, “*Several of the students also wanted to do electives in my area, so I thought that that was very positive*.” “*It’s that feedback that I think also fuels my enthusiasm about the [PBL] formula*”.

While the participants in this study acknowledged the support provided by the PBL trainers and administrative staff, there was general agreement that feedback from faculty was missing and, furthermore, that it would be desirable. One of the tutors indicated that obtaining feedback from someone experienced in PBL would have been preferable to feedback from students. Three of the respondents discounted the importance of feedback from the faculty, indicating that it would not be expected, that “*student feedback was more valuable*” and that the mechanism for how that would take place was unclear.

Three of the respondents included the ‘Thank You’ letter sent to faculty who participated in PBL as a component of the feedback from faculty. Of the ‘Thank You’ letter, one respondent commented *“[It] is great, and very, very important. Very important actually*.”

Timely feedback given to tutors by course coordinators, peer mentors and by students with specific observations and recommendations was identified by the majority of respondents as a factor that would encourage tutors to participate, or to continue to participate, in PBL. *“I think if you’re trying to build a community of committed individuals, the more engaged you are with them the more committed they will be back.”*

### 6. Tutor Rewards

Tutoring in PBL was described as a positive experience by 7 of the 10 tutors and by 2 of the 4 who had chosen not to participate in PBL but who had previous experience as PBL tutors in other settings. One tutor explained:

“It’s given me the opportunity to first hand experience the knowledge base and critical thinking development of the students from first and second year. There is a clear progression of not only their level of knowledge but their level of insight and their critical judgments as it relates to clinical practice. That’s very reassuring and rewarding as a member of the group that is a part of trying to bring them along that process.”

Echoing the value of student contact, another tutor remarked that “*working with the students is tremendous. It’s probably the number one bonus*”.

When specifically asked about the role played by incentives and awards provided by the Faculty of Medicine and Dentistry in the decision to participate in PBL, these were not an expectation on the part of the respondents.

“I think the main rewards are personal.”

“ To me there doesn’t seem to be a lot of rewards except satisfaction.”

“Awards from the Faculty [of Medicine and Dentistry] are irrelevant.”

Monetary awards were discounted as an influential factor by the majority of those interviewed. In contrast, recognition on the faculty member’s annual review (at the department and faculty levels) and a teaching award specific to PBL were acknowledged as highly desirable incentives that would influence decisions to participate. Consistent with this, tutor recognition, clear expectations communicated from the faculty with respect to PBL were the most frequently cited recommendations to recruit and retain tutors in PBL. This included protected time to train and to tutor, equity regarding workload issues, and administrative recognition that PBL is a form of teaching on equal footing with more traditional approaches to teaching. This is reflected in the observation of one tutor who noted “*I still hear around the water cooler from faculty members that it’s not really teaching*”, and by a second respondent that “*a lot of the faculty don’t like the DL style so they haven’t had actual experience doing it*”. Interestingly, recognition of PBL as educational scholarship was identified by one of the non-tutors as an incentive that would have influenced their participation.

### 7. Barriers to Commitment

Virtually all of the respondents cited the time commitment and scheduling issues as concerns regarding participation in PBL. That the schedules for the various blocks of PBL were not available far enough in advance was problematic for tutors who were, in some cases, scheduling events 1 to 2 years into the future. The concentrated time commitment required for tutoring and even for tutor training was a challenge for participants as the PBL schedule requiring 2–3 sessions each week for 5–7 weeks at a time. One participant who had chosen to tutor observed that the decision was based partially on “*utility of the time…. if you see that your role is actually useful in helping the learning process, you are somewhat motivated to make the time*”.

Time challenges were cited as a major reason why tutors chose not to continue to participate in PBL. In addition to the time required for the PBL sessions, tutors commented on the time needed for preparation, to become familiar with cases. Tutors also indicated that despite each department being required to provide tutors for PBL, individuals did not feel well supported by their departments in participating in PBL.

Second only to recognition for service, the area most focused on with respect to recommendations for tutor recruitment and retention was time. Suggestions included regularly letting co-tutors allow individuals to miss sessions or tutor for a part of the course and the incorporation of electronic means such as Skype, again to limit the time commitment.

## Discussion

We anticipate that the findings of this study will be of interest to those responsible for recruiting, training, supporting and retaining faculty members teaching in an academic setting. The factors identified by our participants as contributing to tutor recruitment and retention in problem-based learning are relatively consistent with those identified in the literature regarding motivation, job satisfaction and stress in academia. However, how these factors play out within the context of PBL requires an expansion of our awareness of the motivation of academics in general and serves to inform practices associated with implementation of PBL in a university program.

A number of authors have found that medical faculty enjoy teaching in PBL,^21^ but other studies have noted teacher discomfort in the role of PBL tutor,^22^ and have pointed out the differences between the traditional teacher (‘the sage on the stage’) and the PBL facilitator (‘the guide at the side’).^23^, ^24^ The motivations for teaching observed in this study have also been described in other settings; in a study of doctors teaching in a community setting, intrinsic satisfaction, belonging and recognition were identified as important factors.^25^ Some authors have identified the provision of training, reward, regular feedback and networking opportunities as important factors in retaining PBL tutors.^26^–^28^

It is slowly being recognized that academics are under considerable occupational stress and job dissatisfaction compared to other occupations and settings.^16^, ^29^, ^30^ What motivates faculty to remain engaged in academia is not well understood, however a review by Blackmore and Kandiko^11^ suggests that the following contribute to faculty motivation: (i) internal motivation, including opportunities for growth and development, and (ii) autonomy and independence.

University faculty appear to be influenced less by external reinforcers, such as money, and more by factors of a personal nature. This is evidenced by the continued practice of academics to engage in activities that are not financially profitable.^11^ Our findings support this observation in that money was not considered by our respondents to be a key factor in participation in PBL.

The importance of staff training is underscored by McLean and Van Wyk, “There can be no argument regarding the value of adequate facilitator training at the outset of a new PBL programme”.^28^ Consistent with this, our study found that personal and professional growth and development were identified as reasons to participate in PBL. Participants identified the opportunity to learn a different approach to teaching and learning through the training workshops as well as the practice of giving and receiving feedback.

The debate regarding content-expert tutors may also be approached from the perspective of internal motivation, though admittedly without resolution. Those who are advocates of content-expert tutors may be responding to internal motives, such as feeling that one is making a difference, whereas those who subscribe to the non-content expert facilitator role may be seeking personal growth and development. To be sure, expert versus non-expert tutors is a matter of significant debate within the PBL literature.^31^, ^32^

A point of apparent divergence from internal motivation is the strong message by those interviewed in our study that recognition by administration was important in the recruitment and retention of tutors. However, as Rowley^8^ stresses that “...most [academic] staff have an acute need to feel that their contribution is worthwhile, appreciated, and acknowledged”, suggesting that internal and external motivators may be related.

Finally, the value of building relationships with students, identified in this study as being important to tutors, may also be seen as relating to internal motivation. Whatever the motivation, determining the appropriate incentives appears to be a salient factor in tutor recruitment and retention.^28^

Catano and colleagues^16^ observed that autonomy is central to job satisfaction and reduced stress for academics, having control over for example, how and what one studies and teaches as well as control over time management. The manner in which PBL was implemented in the Faculty of Medicine and Dentistry was such that autonomy was sacrificed for the purpose of consistency. Despite its apparent importance to academics, a lack of autonomy was not identified as a barrier to tutoring for our respondents. In fact, participants valued the attempt to standardize the approach to PBL.

The polarization of respondents around the issue of content expert tutors versus non-content expert tutors may reflect this issue since expertise can be seen to support autonomy. A possible alternative interpretation to explain the acceptance of a non-content expert role is that autonomy for tutors lay in the role of facilitator. Facilitation or tutoring releases the faculty member from the role of delivering content and allows the freedom to explore other components of undergraduate medical training.

It should be noted that two of the study participants indicated an interest in developing cases for PBL, a recommendation also identified by Finucane and colleagues,^33^ which would serve to increase autonomy.

One of the most significant factors identified by our participants and supported by the literature,^33^ which directly impacts autonomy and independence in academia is the resource-intensive nature of PBL, and in particular the time commitment required on the part of tutors. This negative factor appeared to be mitigated substantially by administrative staff who were perceived to be proactive and flexible in addressing the needs of tutors. For example, efforts were made on a case-by-case basis to accommodate tutors by arranging for substitute tutors for individual days and to split courses to allow one person to take on the first part and a second tutor to take on the second half of the course.

### Limitations

While the findings of this study are necessarily limited by the small number of participants and the use of a single semi-structured interview with each participant, the factors identified as being important to recruitment and retention in PBL reflect observations regarding motivations of academics in the larger context of the university. Further investigation is warranted in order to inform practice with respect to the recruitment and retention of tutors in PBL.

### Conclusion

Problem-based learning in higher education is a resource-intensive approach to teaching and learning. The importance of understanding why tutors participate is crucial to the recruitment and retention of tutors, which is in turn fundamental to the success of PBL as part of curriculum. This study examines tutor retention within the larger scope of teacher satisfaction and motivation in medical education, an area not well examined in the current educational research literature. The findings of this study are consistent with the observations of Blackmore and Kandiko^11^ regarding motivation and control of academics in general; however, how these factors are interpreted and addressed within the context of PBL in a medical school was necessarily different than in traditional university approaches to teaching. Identification and strengthening of the factors that promote tutor recruitment and retention may serve to improve PBL initiatives and, furthermore, may increase our understanding of motivation in other aspects of medical education.

## Appendix 1 Interview Questions

1. Why did you initially decide to be a DL tutor?
2. What was it like for you to be a tutor?
3. Are you still a tutor?
4. What factors do you consider when deciding to continue or not to continue being a tutor?
5. If the following factors are relevant to you, can you talk about the role they played in your decision to become or not become a DL tutor?
  - incentives or awards provided by the faculty?
  - support services offered by the faculty?
  - how about tutor training?
  - feed back that you got back from the students?
  - feedback you got back from the faculty?
6. Are there any other factors that were part of your decision that we haven’t talked about yet?
7. Is there anything that could be done to encourage tutors to keep tutoring in PBL?
8. Is there anything else you’d like to add about your experience as a tutor?
